# Magnolol Enhances the Therapeutic Effects of TRAIL through DR5 Upregulation and Downregulation of c-FLIP and Mcl-1 Proteins in Cancer Cells

**DOI:** 10.3390/molecules25194591

**Published:** 2020-10-08

**Authors:** Seon Min Woo, Kyoung-jin Min, Taeg Kyu Kwon

**Affiliations:** 1 Department of Immunology, School of Medicine, Keimyung University, 1095 Dalgubeoldaero, Dalseo-Gu, Daegu 42601, Korea; woosm724@gmail.com (S.M.W.); kjmin@dgmif.re.kr (K.-j.M.); 2 New Drug Development Center, Deagu-Gyeongbuk Medical Innovation Foundation, 80 Chembok-ro, Dong-gu, Daegu 41061, Korea; 3 Center for Forensic Pharmaceutical Science, Keimyung University, Daegu 42601, Korea

**Keywords:** magnolol, TRAIL, DR5, c-FLIP, Mcl-1

## Abstract

Magnolol is a biologically active compound, isolated from the Chinese herb *Magnolia*, that regulates antiproliferative, anticancer, antiangiogenic and antimetastatic activities. We found that magnolol sensitizes TRAIL-induced apoptotic cell death via upregulation of DR5 and downregulation of cellular FLICE-inhibitory protein (c-FLIP) and Mcl-1 in cancer cells, but not in normal cells. Mechanistically, magnolol increased ATF4-dependent DR5 expression at the transcription level, and knockdown of ATF4 markedly inhibited magnolol-induced DR5 upregulation. Silencing DR5 with siRNA prevented combined treatment with magnolol and TRAIL-induced apoptosis and PARP cleavage. Magnolol induced proteasome-mediated Mcl-1 downregulation, while magnolol-induced c-FLIP downregulation was regulated, at least in part, by lysosomal degradation. Our results revealed that magnolol enhanced TRAIL-induced apoptosis via ATF4-dependent DR5 upregulation and downregulation of c-FLIP and Mcl-1 proteins.

## 1. Introduction

Magnolol, a constituent of *Magnolia*, has been used in traditional Chinese herbal medicines for treating gastrointestinal disorders and allergic diseases [1,2]. It possesses diverse biological effects, such as anticancer, antiangiogenetic, antioxidative, antimetastatic and neurotrophic [3,4,5,6]. Magnolol suppresses tumor invasion by inhibiting NF-κB signaling-mediated MMP-9 downregulation in breast cancer, prostate cancer and cholangiocarcinoma [7,8,9]. Multiple studies demonstrate the anticancer function of magnolol. Magnolol induces apoptosis through regulation of the mitochondrial pathway in osteosarcoma, renal cancer and gastric adenocarcinoma [10,11,12]. Magnolol also induces cell cycle arrest and apoptosis via p21- or p27-dependent G2/M phase cell cycle arrest [13,14,15,16]. Moreover, magnolol has a sensitizing effect on chemotherapeutic-agent-mediated cancer cell death. For example, magnolol sensitizes death receptor (DR)-mediated death in non-small cell lung cancer [17]. In addition, magnolol effectively increases the effect of radiation, resulting in the decrease of tumor growth in hepatocellular carcinoma [18]. Inactivation of Akt signaling by magnolol enhances the therapeutic effect of sorafenib through downregulation of antiapoptotic proteins in hepatocellular carcinoma in vitro and in vivo [19]. Therefore, magnolol could be a potential adjuvant that may sensitize the therapeutic efficiency of anticancer drugs.

Tumor necrosis factor-related apoptosis-inducing ligand (TRAIL) can induce caspase-dependent apoptosis of cancer cells by interacting with DRs (DR4 or DR5) [20]. However, because antiapoptotic proteins (Bcl-2, Bcl-xL, Mcl-1, c-FLIP and IAP family) are highly expressed in tumor cells, they reveal TRAIL resistance [21,22]. Therefore, to work out the drawbacks of TRAIL resistance in clinical trials, many researchers have identified TRAIL receptor agonists or chemotherapeutic agents capable of enhancing TRAIL sensitivity [23,24].

Here, we investigated the effects and the underlying mechanisms of magnolol on increasing the sensitivity to TRAIL-mediated cancer cell death.

## 2. Results

### 2.1. Magnolol Enhances Sensitivity to TRAIL in Human Renal Carcinoma Caki-1 Cells

**Figure 1:**
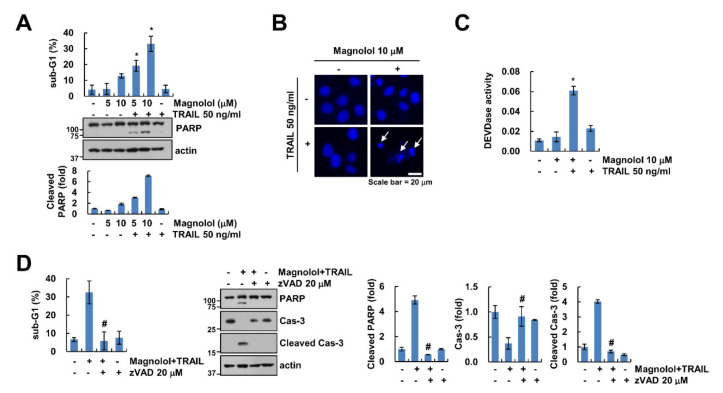
Magnolol induced TRAIL-mediated apoptosis. (**A**) Caki-1 cells were treated with 5–10 μM magnolol alone, 50 ng/mL TRAIL alone or magnolol plus TRAIL for 24 h. (**B**,**C**) Caki-1 cells were treated with 10 μM magnolol alone, 50 ng/mL TRAIL alone or magnolol plus TRAIL for 24 h. DAPI staining (**B**) and DEVDase (caspase-3) activity (**C**) were examined. (**D**) Caki-1 cells were treated with 10 μM magnolol plus 50 ng/mL TRAIL in the presence or absence of 20 μM z-VAD for 24 h. The sub-G1 population and protein expression were detected using flow cytometry (**A**,**D**) and Western blotting (**A**,**D**), respectively. The values in the graph (**A**,**C**,**D**) represent the mean ± SD of three independent experiments. * *p* < 0.01 compared to the control. # *p* < 0.01 compared to the combined treatment with magnolol and TRAIL. White arrows indicate nuclear chromatin condensation.

Previously, many studies demonstrated that magnolol has antitumor effects in many cancer cell lines [10,11,12,13]. Therefore, we examined the effect of a sublethal concentration of magnolol on the sensitivity to TRAIL in renal carcinoma Caki-1 cells. A low concentration of magnolol alone or TRAIL alone did not increase cell death, while co-treatment with magnolol and TRAIL increased the sub-G1 population, PARP cleavage and nuclear chromatin condensation (Figure 1A,B). Moreover, combined treatment activated caspase-3 (Figure 1C). To verify caspase-mediated cell death with the combined treatment of magnolol plus TRAIL, we used a pan-caspase inhibitor, z-VAD. z-VAD significantly inhibited magnolol plus TRAIL-induced PARP and caspase-3 cleavage (Figure 1D). Therefore, these results indicated that magnolol sensitizes TRAIL-induced cancer cell death in a caspase-dependent manner.

### 2.2. Magnolol Downregulates Mcl-1 and c-FLIP Expression and Upregulates DR5 Expression

**Figure 2:**
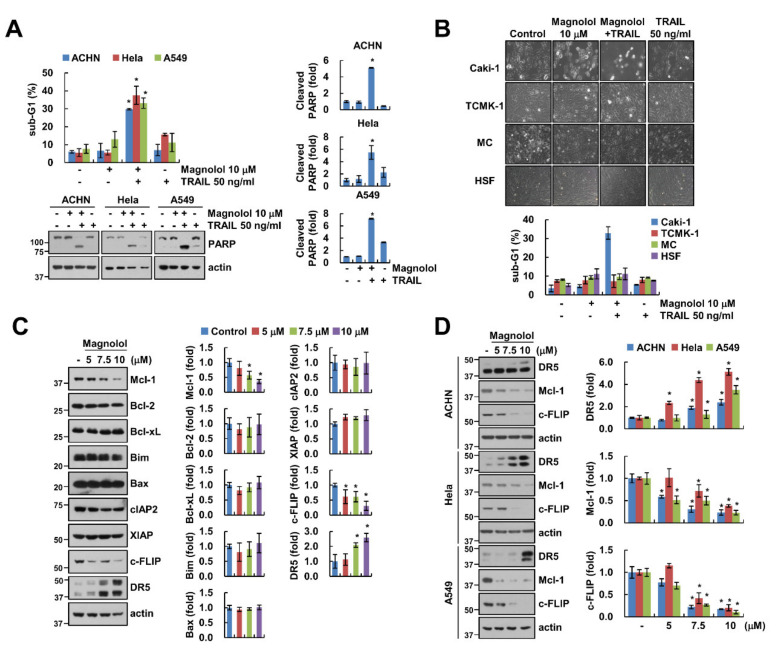
Effect of magnolol on TRAIL-induced apoptosis in other cancer and normal cells. (**A**,**B**) Indicated cancer (**A**) and normal cells (**B**) were treated with 10 μM magnolol alone, 50 ng/mL TRAIL alone or magnolol plus TRAIL for 24 h. (**C**,**D**) Caki-1 (**C**) and indicated cancer cells (**D**) were treated with various concentrations of magnolol for 24 h. The sub-G1 population and protein expression were detected using flow cytometry (**A**,**B**) and Western blotting (**A**,**C**,**D**), respectively. Cell morphology was examined using interference light microscopy (**B**). The values in graph (**A**–**D**) represent the mean ± SD of three independent experiments. * *p* < 0.01 compared to the control.

We investigated the sensitizing effect of magnolol on TRAIL-induced apoptosis in various cancer cells and normal cells. Combined treatment induced apoptosis in other renal carcinoma ACHN, cervical cancer Hela and lung cancer A549 cells (Figure 2A). However, cell death of normal kidney cells (TCMK-1, MC) and human skin fibroblasts (HSF) was not induced by the combined treatment of magnolol plus TRAIL (Figure 2B). Next, we checked the expression levels of apoptosis-related proteins under the effect of magnolol. Magnolol decreased Mcl-1 and c-FLIP expression and increased DR5 expression. However, other proteins (Bcl-2, Bcl-xL, Bim, Bax and IAP family proteins (cIAP2 and XIAP)) were not changed by magnolol treatment (Figure 2C). We then examined whether the effect of magnolol on DR5, Mcl-1 and c-FLIP expression patterns is restricted to Caki-1 cells. We found a similar expression pattern of these proteins in ACHN, Hela and A549 cells (Figure 2D).

### 2.3. ATF4-Mediated DR5 Upregulation Contributes to Magnolol Plus TRAIL-Induced Apoptosis

**Figure 3:**
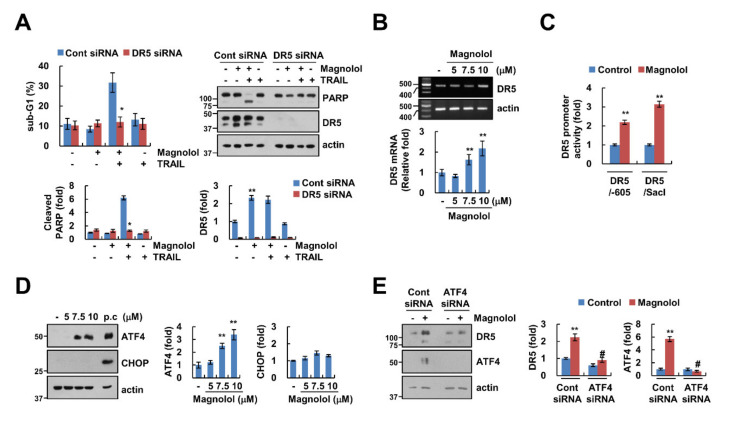
ATF4-dependent DR5 upregulation by magnolol was involved in TRAIL-induced apoptosis. (**A**) Caki-1 cells were transfected with control (Cont) or DR5 siRNA and were treated with 10 μM magnolol alone, 50 ng/mL TRAIL alone or magnolol plus TRAIL for 24 h. (**B**) Caki-1 cells were treated with various concentrations of magnolol for 24 h. The levels of mRNA were examined using RT-PCR and quantitative PCR (qPCR). (**C**) Caki-1 cells were transiently transfected with DR5 (-605) or DR5 (SacI) promoter and incubated with 10 μM magnolol for 24 h. The cells were lysed, and the luciferase activity was measured as described in Materials and Methods. (**D**) Caki-1 cells were treated with various concentrations of magnolol for 9 h. (positive control (p.c); 2 μM brefeldin A). (**E**) Caki-1 cells were transfected with Cont or ATF4 siRNA and were treated with 10 μM magnolol for 9 h. The sub-G1 population and protein expression were detected using flow cytometry (**A**) and Western blotting (**A**,**D**,**E**), respectively. The values in graph (**A**–**E**) represent the mean ± SD of three independent experiments. * *p* < 0.01 compared to the combined treatment with magnolol and TRAIL in control siRNA. ** *p* < 0.01 compared to control. # *p* < 0.01 compared to magnolol in control siRNA.

To examine the involvement of DR5 in magnolol-mediated TRAIL sensitization, we performed a knockdown assay using siRNA. The knockdown of DR5 markedly attenuated combined treatment-induced apoptosis (Figure 3A). Magnolol treatment induced upregulation of DR5 mRNA levels and DR5 promoter activity (Figure 3B,C). Therefore, these data suggest that magnolol induced DR5 upregulation at the transcription level.

Previous studies reported that ATF4 and CHOP, well-known marker proteins of ER stress, work as transcription factors capable of regulating DR5 expression [25,26]. Therefore, we investigated expression levels of ATF4 and CHOP proteins. Interestingly, magnolol induced upregulation of ATF4 expression, but not CHOP expression (Figure 3D). We further examined whether ATF4 was involved in magnolol-mediated DR5 upregulation. As shown in Figure 3E, silencing ATF4 abolished the upregulation of DR5 by magnolol. Therefore, these results suggested that magnolol-induced DR5 upregulation contributes to TRAIL sensitization through the increase of ATF4 expression.

### 2.4. Downregulation of c-FLIP and Mcl-1 by Magnolol Is Involved in TRAIL-Mediated Apoptosis

**Figure 4:**
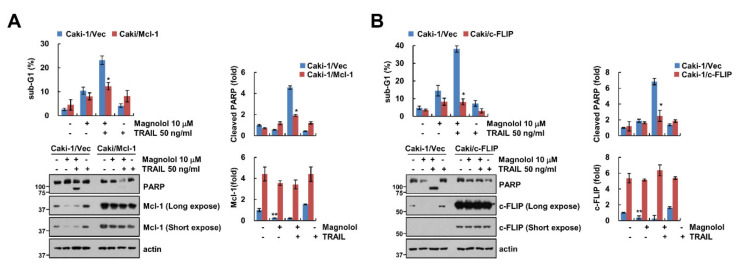
Overexpression of Mcl-1 and c-FLIP attenuated magnolol plus TRAIL-induced apoptosis. (**A**,**B**) Vector cells, Mcl-1- (**A**), and c-FLIP-overexpressing cells (**B**) were treated with 10 μM magnolol alone, 50 ng/mL TRAIL alone or magnolol plus TRAIL for 24 h. The sub-G1 population and protein expression were detected using flow cytometry and Western blotting, respectively (**A**,**B**). The values in graph (**A**,**B**) represent the mean ± SD of three independent samples. * *p* < 0.01 compared to combined treatment with magnolol and TRAIL in Caki-1/Vec. ** *p* < 0.01 compared to control.

As shown in Figure 2C, magnolol induced downregulation of Mcl-1 and c-FLIP expression; we investigated whether these proteins are associated with magnolol-mediated TRAIL sensitization. As expected, ectopic expression of Mcl-1 and c-FLIP markedly attenuated the combined treatment-induced sub-G1 population and PARP cleavage (Figure 4A,B). These data indicated that downregulation of Mcl-1 and c-FLIP had a crucial role in augmenting TRAIL-mediated apoptosis with magnolol.

### 2.5. Magnolol Induces Downregulation of Mcl-1 and c-FLIP Protein Expression at the Post-Translation Level

**Figure 5:**
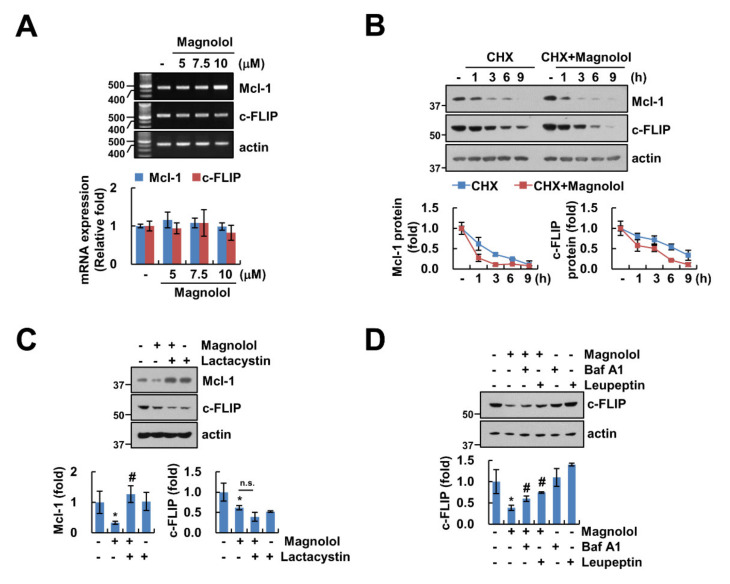
Magnolol induced downregulation of Mcl-1 and c-FLIP proteins at the post-translational level. (**A**) Caki-1 cells were treated with various concentrations of magnolol for 24 h. mRNA expression was detected using RT-PCR and qPCR. (**B**) Caki-1 cells were treated with 10 μM magnolol in the presence or absence of 20 μM CHX for the indicated time kinetics. Band intensity was quantified using Image J. (**C**,**D**) Caki-1 cells were treated with 10 μM magnolol in the presence or absence of 2.5 μM lactacystin (**C**), 5 nM bafilomycin A (Baf A1) (**D**), and 10 μM leupeptin (**D**) for 24 h. Protein expression was detected using Western blotting (**B**–**D**). The values in graph (**A**–**D**) represent the mean ± SD of three independent experiments. * *p* < 0.01 compared to the control. # *p* < 0.01 compared to magnolol. n.s. = no significant.

Magnolol downregulated Mcl-1 and c-FLIP protein levels, but these mRNAs were not altered by magnolol treatment (Figure 2C, Figure 5A). To explore how to regulate magnolol-mediated Mcl-1 and c-FLIP, we used an inhibitor of protein biosynthesis, cycloheximide (CHX), for measuring protein stability. As shown in Figure 5B, compared to CHX alone, magnolol inhibited the stability of Mcl-1 and c-FLIP. Magnolol-induced downregulation of Mcl-1 and c-FLIP was reversed, respectively, by proteasome inhibitors (lactacystin) and lysosome inhibitors (bafilomycin A1 (Baf A1) and leupeptin) (Figure 5C,D). Therefore, these results suggested that magnolol-induced downregulation of Mcl-1 and c-FLIP proteins was regulated at the post-translational level.

### 2.6. Generation of ROS Is Not Associated with Magnolol Plus TRAIL-Induced Apoptosis

**Figure 6:**
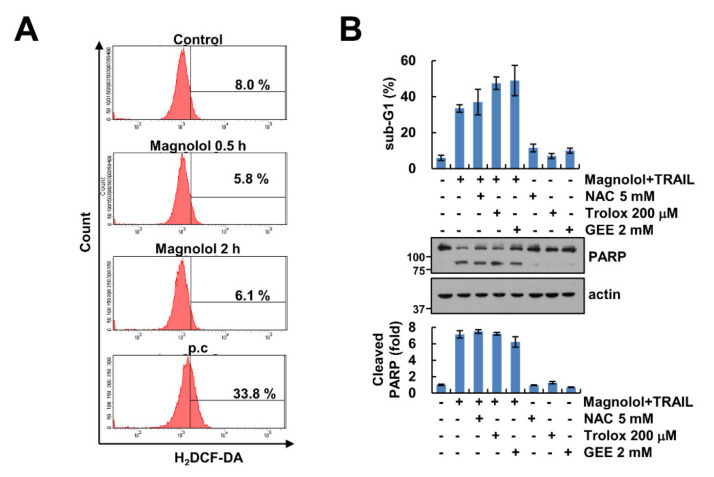
Involvement of ROS in magnolol plus TRAIL-induced apoptosis. (**A**) Caki-1 cells were treated with 10 μM magnolol for 0.5 and 2 h. Thioridazine was used as positive control (p.c). Caki-1 cells were stained with H_2_DCF-DA dye. Fluorescence was detected using flow cytometry. (**B**) Caki-1 cells were treated with 10 μM magnolol plus 50 ng/mL TRAIL in the presence or absence of 5 mM NAC, 200 μM trolox or 2 mM GEE for 24 h. The sub-G1 population and PARP cleavage were detected using flow cytometry and Western blotting, respectively. The values in graph (**B**) represent the mean ± SD of three independent samples.

Previous studies showed that magnolol generates intracellular ROS, resulting in increased apoptosis [11,13]. We examined the involvement of ROS in magnolol-mediated TRAIL sensitization. However, magnolol did not increase intracellular ROS in Caki-1 cells (Figure 6A), and combined treatment-induced apoptosis and PARP cleavage were not inhibited by ROS scavengers (NAC, trolox and GEE) (Figure 6B). These results suggested that ROS was not associated with apoptosis induced by combined treatment with magnolol plus TRAIL.

## 3. Discussion

In this study, we suggested that magnolol could work as a sensitizer to TRAIL in cancer cells. We found involvement of magnolol-mediated DR5, Mcl-1 and c-FLIP regulation in TRAIL sensitization. Magnolol increased ATF4-dependent DR5 expression at the transcription level and downregulation of Mcl-1 and c-FLIP expression at the post-translation level. Furthermore, knockdown of DR5 or overexpression of Mcl-1 and c-FLIP markedly blocked magnolol plus TRAIL-induced apoptosis.

Previously, Liu et al. reported magnolol as a novel HDAC class I inhibitor that hyperacethylates histones H3 and H4, especially the H3K27 site within the DR5 promoter, resulting in an increase of TRAIL-induced apoptosis [17]. We found that magnolol increased DR5 mRNA expression. Transcription factor ATF4 was involved in magnolol-mediated DR5 upregulation (Figure 3B,E). Lin et al. reported that magnolol induced Bcl-2 downregulation and Bax activation in colon and liver cancer cells [27]. However, magnolol did not alter Bcl-2 and Bax expression in our system (Figure 2C). This contradiction was believed to be due to the difference of cell contexts and drug concentration. Whereas Lin et al. used a high concentration of magnolol (60 μM), we used a low concentration (10 μM). Recently, Wang et al. reported that magnolol (30 μM) exerted anticancer activity via induction of CHOP-mediated ER stress in hepatocellular carcinoma HepG2 cells, and inhibition of CHOP abrogates magnolol-induced apoptosis [28]. In our results, magnolol (10 μM) did not induce CHOP expression, but another ER stress marker protein, ATF4, was induced by magnolol (Figure 3D). We found that magnolol increased DR5 expression at the transcription level through ATF4 upregulation, instead of CHOP upregulation (Figure 3D). Moreover, knockdown of ATF4 markedly blocked DR5 upregulation in magnolol-treated cells (Figure 3E). Therefore, these data indicated that upregulation of DR5 using a low concentration of magnolol was influenced by the increase of ATF4 expression.

In previous studies, magnolol induced downregulation of Mcl-1 and c-FLIP expression, and inhibited tumor progression of colorectal cancer [29]. However, those studies did not examine the molecular mechanisms of these proteins. We also showed the downregulation of Mcl-1 and c-FLIP protein expression, but not mRNA (Figure 2C, Figure 5A). Activation of proteasome is an important function in protein degradation in the ubiquitin proteasome system (UPS) [30]. We demonstrated that UPS was involved in Mcl-1 and c-FLIP degradation with magnolol treatment. However, magnolol-induced Mcl-1 downregulation was prevented by a proteasome inhibitor, whereas lysosome inhibitors disrupted magnolol-mediated c-FLIP downregulation (Figure 5C,D). Therefore, these data indicated that magnolol may differently modulate protein expression levels of Mcl-1 and c-FLIP. Because E3 ligases and deubiquitinases are associated with protein degradation in the UPS system, we need to further study the involvement of the molecular mechanism of magnolol-induced downregulation of Mcl-1 and c-FLIP.

Collectively, we showed that magnolol sensitized cancer cells to TRAIL-induced apoptosis through ATF4-dependent DR5 upregulation, proteasome-mediated Mcl-1 downregulation and lysosome-mediated c-FLIP downregulation. Therefore, we demonstrated that modulation of DR5, Mcl-1 and c-FLIP with magnolol played a critical role in the enhancement of TRAIL sensitization.

## 4. Materials and Methods

### 4.1. Cell Lines and Culture

All cancer cells (Caki-1, ACHN, A549 and Hela) and TCMK-1 cells were obtained from American Type Culture Collection (Manassas, VA, USA). Human mesangial cells (MCs) were purchased from Lonza (Basel, Switzerland). Normal human skin fibroblast (HSF) cells were provided by Korea Cell Line Bank (Seoul, Korea). Cells were grown in an appropriate medium supplemented with 10% fetal bovine serum (FBS) (Welgene, Gyeongsan, Korea), 1% penicillin–streptomycin and 100 μg/mL gentamycin (Thermo Fisher Scientific, Waltham, MA, USA). To construct stable cell lines, Caki-1 cells were transfected using Lipofectamine^TM^ 2000 (Invitrogen, Carlsbad, CA, USA) with pcDNA3.1(+)/Mcl-1, pcDNA3.1(+)/c-FLIP or pcDNA3.1(+) vector plasmids and selected using 700 μg/mL G418 (Invitrogen, Carlsbad, CA, USA). Immunoblot analysis was performed to examine protein expression [31].

### 4.2. Reagents and Antibodies

Sigma Chemical Co. provided magnolol, cycloheximide, bafilomycin A1, leupeptin and anti-actin (St. Louis, MO, USA). R&D Systems supplied recombinant human TRAIL and z-VAD (Minneapolis, MN, USA). Enzo Life Sciences provided lactacystin, anti-pro-caspase-3 and anti-c-FLIP (Ann Arbor, MI, USA). Santa Cruz Biotechnology provided anti-Mcl-1, anti-Bcl-2, anti-cIAP2 and anti-ATF4 (St. Louis, MO, USA). Cell Signaling Technology supplied anti-PARP, anti-cleaved caspase-3, anti-Bcl-xL, anti-DR5 and anti-CHOP (Beverly, MA, USA). BD Biosciences provided anti-Bim and anti-XIAP (San Jose, CA, USA).

### 4.3. FACS Analysis and DAPI Staining

To detect apoptosis, we used two methods. For FACS analysis, cells were harvested and suspended in 100 μL of phosphate-buffered saline and added to 200 μL of 95% ethanol. Then, cells were incubated in a 1.12% sodium citrate buffer containing RNase at 37 °C for 30 min, added to 50 μg/mL propidium iodide and analyzed using a BD Accuri™ C6 flow cytometer (BD Biosciences, San Jose, CA, USA). To check nuclei condensation, cellular nuclei cells were stained with a 300 nM 4′, 6′-diamidino-2-phenylindole solution (Roche, Mannheim, Germany), and we viewed fluorescence images using fluorescence microscopy (Carl Zeiss, Jena, Germany).

### 4.4. Western Blotting

Cells were lysed in a lysis buffer (20 mM HEPES and 0.5% Triton X-100, pH 7.6) and separated using 10% SDS-PAGE. To analyze protein expression, proteins were transferred to nitrocellulose membranes (GE Healthcare Life Science, Pittsburgh, PO, USA) and checked using Immobilon Western Chemiluminescent HRP Substrate (EMD Millipore, Darmstadt, Germany).

### 4.5. DEVDase (Caspase-3) Activity

To measure DEVDase activity, cells were harvested and incubated with a reaction buffer containing acetyl-Asp-Glu-Val-Asp p-nitroanilide (Ac-DEVD-pNA) substrate, as previously mentioned [32].

### 4.6. Knockdown of Genes Using siRNA

GFP (control) and DR5 siRNA duplexes were purchased from Bioneer (Daejeon, Korea) and Invitrogen (Carlsbad, CA, USA), respectively. ATF4 siRNA duplexes were purchased from Santa Cruz Biotechnology (St. Louis, MO, USA). For the knockdown of gene by siRNA, Lipofectamine^®^ RNAiMAX Reagent (Invitrogen, Carlsbad, CA, USA) was used in Caki-1 cells. Immunoblot analysis was performed to examine protein expression.

### 4.7. Reverse Transcription Polymerase Chain Reaction (RT-PCR) and Quantitative PCR (qPCR)

Total RNA was isolated using TriZol reagent (Life Technologies, Gaithersburg, MD, USA), and cDNA was prepared using M-MLV reverse transcriptase (Gibco-BRL, Gaithersburg, MD, USA). For PCR, we used Blend Taq DNA polymerase (Toyobo, Osaka, Japan) with primers targeting DR5, c-FLIP, Mcl-1 and actin. For qPCR, SYBR Fast qPCR Mix (Takara Bio Inc., Shiga, Japan) was used, and reactions were performed on a Thermal Cycler Dice^®^ Real Time System III (Takara Bio Inc., Shiga, Japan). We calculated the threshold cycle number (Ct) of each gene using actin as the reference gene, and we reported the delta-delta Ct values of the genes. The used primers were referred to in previous studies [33].

### 4.8. Promoter Activity Assay

The method using this assay was described in our previous study [34]. Briefly, cells were transfected with DR5 (-605) or DR5 (SacI) promoter-constructs using Lipofectamine™2000 (Invitrogen, Carlsbad, CA, USA) and harvested in a lysis buffer (25 mM Tris-phosphate pH 7.8, 2 mM EDTA, 1% Triton X-100, and 10% glycerol). The supernatants were used to measure luciferase activity according to the manufacturer’s instructions (Promega, Madison, WI, USA).

### 4.9. Measurement of Reactive Oxygen Species

Intracellular accumulation of ROS was determined using the fluorescent probes 2, 7-dichlorodihydrofluorescein diacetate (H_2_DCF-DA). The cells were treated with magnolol and stained with the H_2_DCF-DA fluorescent dye for an additional 10 min. Then, the cells were trypsinized and resuspended in PBS, and fluorescence was measured at specific time intervals using a FACS Canto II (BD Biosciences, San Diego, CA, USA).

### 4.10. Statistical Analysis

The data were analyzed using one-way ANOVA and post-hoc comparisons (Student–Newman–Keuls) in SPSS software (SPSS Inc., Chicago, IL, USA). The values represented the mean ± SD of at least three independent experiments.

## 5. Conclusions

Magnolol sensitized cancer cells to TRAIL-induced apoptosis through ATF4-dependent DR5 upregulation and proteasome-mediated Mcl-1 and c-FLIP downregulation. Therefore, we demonstrated that modulation of DR5, Mcl-1 and c-FLIP by magnolol played a critical role in the enhancement of TRAIL sensitization.
